# Design and methods for testing a simple dietary message to improve weight loss and dietary quality

**DOI:** 10.1186/1471-2288-9-87

**Published:** 2009-12-30

**Authors:** Philip A Merriam, Yunsheng Ma, Barbara C Olendzki, Kristin L Schneider, Wenjun Li, Ira S Ockene, Sherry L Pagoto

**Affiliations:** 1 Division of Preventive and Behavioral Medicine, Department of Medicine, University of Massachusetts Medical School, Worcester, MA, USA; 2 Division of Cardiovascular Medicine, Department of Medicine, University of Massachusetts Medical School, Worcester, MA, USA

## Abstract

**Background:**

The current food pyramid guidelines have been criticized because of their complexity and the knowledge required for users to understand the recommendations. Simplification of a dietary message to focus on a single key aspect of dietary quality, e.g., fiber intake, may make the message much easier to comprehend and adhere, such that respondents can achieve greater weight loss, better dietary quality and overall metabolic health.

**Methods and design:**

This is a randomized controlled clinical trial with two equal sized arms. In total, 240 obese adults who meet diagnostic criteria for the metabolic syndrome will be randomized to one of the two conditions: 1) a high fiber diet and 2) the American Heart Association (AHA) diet. In the high fiber diet condition, patients will be given instruction only on achieving daily dietary fiber intake of 30 g or more. In the AHA diet condition, patients will be instructed to make the several dietary changes recommended by the AHA 2006 guidelines. The trial examines participant weight loss and dietary quality as well as changes in components of the metabolic syndrome, inflammatory biomarkers, low-density lipoprotein cholesterol levels, insulin levels, and glycosolated hemoglobin. Potential mediators, i.e., diet adherence and perceived ease of the diet, and the intervention effect on weight change will also be examined.

**Discussions:**

The purpose of this paper is to outline the study design and methods for testing the simple message of increasing dietary fiber. If the simple dietary approach is found efficacious for weight loss; and, improves dietary quality, metabolic health, and adherence, it might then be used to develop a simple public health message.

**Trial registration:**

NCT00911885

## Background

Metabolic syndrome affected nearly 27% of US adults in 2006, and due to rising obesity rates, the prevalence of metabolic syndrome is likely much higher in 2009. Metabolic syndrome is strongly associated with poor dietary quality [1], and treating it is one of the keys to the prevention of cardiovascular disease (CVD) [2,3] and diabetes [4,5]. Research is needed on interventions that effectively treat metabolic syndrome, preventing its advance to the physical, mental, and financial costs of CVD and diabetes.

The impact of public health campaigns is maximized when a health message is simple and easy to understand [6]. Dietary guidelines like those put forth by the American Heart Association (AHA), US Department of Agriculture (USDA), and the American Diabetes Association (ADA) [7-9] are based on research and are in the interest of public health but are also complex involving multiple macronutrients, each with differing recommended portion sizes and daily servings. A healthy diet is key to CVD and diabetes prevention but we are lacking a simple, effective public health message to improve the American diet.

A simple dietary message is only possible to the extent that one area of diet is identified that, on the one hand, has a strong impact on overall dietary quality and disease prevention, and on the other hand, is associated with good adherence and acceptability. One possible area of diet that could meet these two criteria is dietary fiber. Dietary fiber has been demonstrated to be a useful component of weight loss and weight loss maintenance [10-12] and it acts directly on several aspects of the metabolic syndrome including decreasing waist circumference and body weight, glucose and lipid homeostasis, and improving hypertension and insulin control [13,14].

### Body weight and waist circumference

Observational and review studies have indicated an inverse relationship between dietary fiber intake, body weight and waist-to-hip ratio ([10-12] with several relatively short-term intervention studies further supporting the relationship between a high fiber diet and moderate reductions of body weight and waist circumference [15-17]. Epidemiologic studies support a strong negative association between dietary fiber intake and obesity. However, a limited number of clinical trials have been conducted directly associating a simple dietary fiber message with metabolic components, including the mechanism by which fiber acts. Howard and colleagues concluded from 12 published intervention studies that under conditions of fixed energy intake, an increase in dietary fiber intake increased post-meal satiety and decreased subsequent hunger [10]. When energy intake is ad libitum, increasing consumption of dietary fiber is associated with weight loss via a decrease in energy intake. A review by Lairon also supports these results [12]. It is proposed that high-fiber foods promote satiety through delayed gastric emptying, increased food volume, and increased chewing, which attenuates the return of hunger [18,19], and leads to decreased energy intake. In fact, Pereira and colleagues reviewed 27 clinical studies and concluded that most studies showed that an increased fiber intake correlates with a reduced energy intake of 10% [18]. In addition, fiber decreases the absorption efficiency of the small intestine ([11].

### Insulin resistance and hypertension

An increased intake of total fiber is inversely associated with markers of insulin resistance and reduced diabetes risk [20-22]. The Insulin Resistance Atherosclerosis Study showed that dietary fiber was significantly associated with insulin sensitivity, fasting insulin, body mass index (BMI), and waist circumference [22]. Similarly, in the Inter99 study, intake of dietary fiber was inversely associated insulin resistance estimated using the homeostasis model assessment of insulin resistance (HOMA-IR) [21]. In addition, soluble dietary fiber has been reported to reduce postprandial glucose levels and to improve insulin sensitivity [23,24]. These findings support the recommendation to increase intake of fiber-rich carbohydrates to prevent insulin resistance [25]. Clinical trials indicate that a diet high in fiber decreases blood pressure in hypertensive and obese individuals [26,27]. Since insulin resistance with compensatory hyperinsulinemia has been named a major pathogenic vehicle for the development of hypertension [28], reducing insulin resistance through increasing dietary fiber intake may help treat or prevent hypertension. In addition, increasing dietary fiber intake promotes weight loss and deters weight gain, both of which would likely have a large impact on the prevention and burden of hypertension.

### Inflammation biomarkers

Observational studies draw a significant link between dietary fiber intake and reduced levels of C-reactive protein (CRP) [29-32]. In a small clinical trial of 28 subjects, King and colleagues demonstrated that increasing fiber lowered the levels of CRP [33]. We have discussed four possible mechanisms between dietary fiber and inflammation in our two recent publications [31,32]. First, dietary fiber decreases lipid oxidation, which in turn is associated with decreased inflammation [34]. Second, dietary fiber supports normal bowel flora as part of an optimal intestinal environment, which helps to prevent inflammation [34]. Third, dietary fiber may inhibit inflammation through its beneficial effects on glycemic control [35,36]. Finally, diets high in fiber may increase plasma adiponectin concentrations in diabetic patients: adiponectin has been found to have profound anti-inflammatory effects [37].

### Glycemic and lipid control

In a crossover design of 11 patients with metabolic syndrome, patients supplemented a high-carbohydrate diet with soluble fiber for 3 weeks [38]. Results indicate that a high fiber diet improves glycemic control, total and LDL cholesterol, while triglycerides and HDL cholesterol remained unchanged.

Our preliminary pilot study data suggest that simply focusing on increasing dietary fiber is equivalent or better than a low-saturated fat message at inducing clinically significant improvement of dietary quality, and may be superior for long-term adherence [39]. Participants were randomized to receive either a simple message to increase fiber intake, a simple message to reduce saturated fat intake, or a combination to increase fiber and reduce saturated fat. At 3 months, participants in the fiber arm increased their fiber intake by 44% and vicariously reduced their saturated fat intake by 25%. Participants in the saturated fat arm reduced their saturated fat intake by 25% but their fiber intake only changed by 2%. These changes were maintained at 6 months. The dual message arm did not show any significant improvement over the dietary fiber arm alone. Patients in the high fiber group lost 7 lbs at 3 months, and 10 lbs at 6 months. The single message of increasing dietary fiber might be more acceptable by encouraging *increases *in intake of particular foods as opposed to depriving messages about eliminating foods. When asked at 3 months about confidence in adhering to the study diet, 85% of participants in the high fiber group felt very or extremely confident they could adhere to the recommendation, while only 50% of participants in the decrease saturated fat condition and 40% of participants in the combination condition felt this way.

We hypothesize that a simplified dietary recommendation focusing on a single aspect of dietary quality - fiber intake - will facilitate weight loss, and improve both metabolic health and overall dietary quality. Such a simplified dietary advisory is easier to follow, and may have beneficial collateral effects on other areas of diet (e.g., reduced caloric and saturated fat intake, and increased intake of protective foods). Such a simple message, if found efficacious for weight loss, metabolic health and dietary quality in a clinical setting, might then be an ideal message for public health settings.

### Research Goals

The overall goal of the present study is to compare the efficacy of two dietary intervention approaches on weight loss and improving dietary quality among patients with the metabolic syndrome. One approach is complex and the other is simple. The two approaches are: 1) the AHA Dietary Guidelines [40] that is currently recommended to patients with the metabolic syndrome [41,42]; and 2) a high fiber diet that provides instruction on a single area of diet, fiber. Secondary research goals include examining changes in components of the metabolic syndrome, inflammatory markers, low-density lipoprotein (LDL-C) cholesterol levels, insulin levels, and glycosolated hemoglobin (HbA1c). We hypothesize that the high fiber condition will significantly improve overall diet quality and metabolic health over the AHA condition. Additionally, potential mediators (i.e., adherence and perceived ease of the diet) of the intervention effect at 12 months will also be examined.

## Methods/Design

### Study Design

The study protocol was approved by the University of Massachusetts Medical School's (UMMS) Institutional Review Board. Two hundred forty adults (50% female) who meet diagnostic criteria for the metabolic syndrome will be randomized to the high fiber condition or the AHA diet condition. The study was funded by the National Heart, Lung and Blood Institute.

### Subject Eligibility Criteria

To be eligible for the study, an individual must: 1) Meet diagnostic criteria for the metabolic syndrome [43]; 2) Be interested in losing weight and have a BMI 30-40 kg/m^2^; 3) Be between 21 to 70 years old; 4) Have a telephone in the home or easy access to one; 5) Provide informed consent; 6) Have physician approval to participate in the study; 7) Be a non-smoker (given nicotine's effect on weight suppression, on HDL-C, and smoking cessation's effect weight gain); and, 8) Be able to speak and read English.

An individual will be excluded from participation if he/she:1) Has clinically diagnosed diabetes, or a fasting blood sugar of ≥ 126 mg/dl; 2) Had an acute coronary event within the previous 6 months; 3) Is pregnant or lactating; 4) Is a woman with polycystic ovary syndrome [44]; 5) Plans to move out of the area within the 12-month study period; 6) Has a diagnosis of a medical condition that precludes adherence to study dietary recommendations (e.g., Crohn's disease, ulcerative colitis, active diverticulitis, renal disease); 7) Has elevated depression or suicidal ideation; 8) Is following a low-carbohydrate, high-fat dietary regimen such as the Atkins' Diet [45]; 9) Is participating in any current weight loss program; 10) Has had bariatric surgery or is currently using weight loss medication; or, 11) Has been diagnosed with an eating disorder (bulimia nervosa or binge eating).

### Recruitment

Study recruitment began in May 2009. Recruitment strategies include: posting study recruitment fliers at the University of Massachusetts Medical School (UMMS), local public libraries and churches; announcements on the UMMS intranet; recruitment ads in the local newspapers and on Craigslist; and targeted direct mailings. All IRB approved posters and advertisements include a phone number that individuals can call. Potential subjects responding to study advertisements receive an explanation of the study and are screened via telephone using a brief questionnaire focused on inclusion and exclusion criteria. This information is retained in a database with Lotus Notes/IBM tracking system software (Lotus Notes R5.0.11^®^) developed specifically for this study. When an individual is found to be pre-eligible for the study and expresses interest in participating, a screening appointment is then scheduled.

The University is currently updating its clinical data system and once it is complete, we will be able to identify patients who are eligible using a primary care patient population database. A HIPAA-IRB waiver will be obtained to identify eligible patients.

### Study Measures at Screening Visit, Baseline Visit and Patient Follow-up

At the screening visit, a screening consent form is reviewed and signed. The Cholestech LDX System™ is used to measure HDL-C, triglycerides, and glucose from a fasting fingerstick with results produced within 10 minutes. Individuals are asked to fast 12-hours prior to the appointment. Patients have their height and weight measured in stocking feet via an electronic digital scale (Scale-Tronix, Carol Stream, Illinois, Model 5002 Stand-On Scale). Waist is measured twice at the narrowest part of the torso (or a site between the lower rib and crest of the hipbone). Blood pressure is also measured two times: initially after sitting quietly for 10 min, then again after 2 minutes using a Dinamap XL ^® ^automated BP monitor (Critikon). The Center for Epidemiological Studies-Depression Scale (CES-D)[46,47] is administered to assess for depression. Individuals with CES-D >= 21, indicating severe depressive symptoms [48], are excluded from participation. Medical clearance is then requested from the primary care physician, and individuals found to be eligible are invited back for a baseline visit.

At the baseline visit, a second study consent form is reviewed with the research assistant and signed. A fasting blood sample, anthropometric measures, and medication and supplement information are collected, which will again be assessed at the 3-, 6-, and 12-month visits. A questionnaire packet assessing demographic information and psychosocial variables is completed at baseline, 3-, 6- and 12-month visits. Three 24-hour diet and physical activity recalls are collected within a three-week window at baseline, 6- and 12-month visits to determine individual dietary and physical activity change, and one 24-hour recall is collected at 3 months to determine group differences. The 3-month visit assessment will measure short-term changes in body weight and metabolic syndrome indicators, however, inflammatory markers, insulin, and HbA1c will not be measured. Table 1 includes a complete list of study measures and the timing of these measures. Patients receive a stipend of $10 at baseline and the 3 month assessment; $20 at the 6-month assessment; and $40 at the 12 month assessment.

**Table 1:** Measurement Schedule

	Timepoint			
**Measure**	**Baseline**	**3 mos**	**6 mos**	**12 mos**

**Physiological measures:**				
• Blood glucose	**●**	**●**	**●**	**●**
• Glycosylated hemoglobin	**●**		**●**	**●**
• Blood pressure	**●**	**●**	**●**	**●**
• Insulin	**●**		**●**	**●**
• Inflammatory markers (hs-CRP, Il-6, TNF-a)	**●**		**●**	**●**
• Serum lipids	**●**	**●**	**●**	**●**
• Body habitus measures (height, weight, and waist circumference)	**●**	**●**	**●**	**●**
				
**Diet, physical activity, medication use, and psychological variables:**				
• 24-hr dietary recall (3 times at each timepoint)	**●**	**●**	**●**	**●**
• 24-hr physical activity recall (3 times at each timepoint except 1 recall only at 3 mths)	**●**	**●**	**●**	**●**
• Medication use	**●**		**●**	**●**
• Depressive symptoms (CES-D)	**●**		**●**	**●**
• Quality of life (SF-36)	**●**		**●**	**●**
• Social desirability	**●**			
				
**Patient Characteristics**				
• Socio-demographic variables	**●**			
• Medical history	**●**			
				
**Process Variables**				
• Retention rate				**●**
• Session attendance				**●**
• Intervention acceptability			**●**	**●**
• Self-efficacy, attitudes, social support, perceived barriers related to dietary changes	**●**	**●**	**●**	**●**

### Randomization

After providing informed consent and completing the baseline assessment, participants are randomized to one of the two diet conditions. Participants are stratified by gender, age (in deciles), and BMI categories (30-34.9, and >= 35-40 kg/m^2^). Within each strata, participants are randomized to the two conditions in randomly permuted blocks of size 3 and 6 using the ralloc program in Stata [49] to ensure that the distributions of gender, age, and BMI are similar between two conditions. The randomization is carried out by the project director who does not interact with participants.

### Intervention

The proposed intervention will consist of 5 sessions during a 3-month intensive phase (1 group session in the 1^st ^month, one individual and one group session during the 2nd month, and biweekly sessions in month 3), and a 9-month maintenance phase of 5 group sessions (during the 4^th^, 5^th^, 7^th^, 9^th^, and 11^th ^months) and one individual session (at the 12^th ^month) for a total of 11 sessions. Individual sessions will be offered at different days and times to accommodate participant schedules.

Patients will have received a diet manual at the first group visit, containing intervention contents by session, home activity worksheets, resources, recipes, and selected menus with nutrition information from restaurants (either AHA or dietary fiber oriented). At the next individual nutrition consultation, an assessment of lifestyle, current dietary habits, challenges to dietary changes and specific nutrition needs, and individualized study goals are reviewed by a registered dietitian. Patients begin tracking their dietary intake in preparation for the third group visit. All group and individualized sessions will be conducted by a registered dietitian initially randomly assigned and trained to the study condition, and will focus on reviewing progress and setting new goals to support achievements. Each session will address any challenges to adherence and facilitate progress toward the patient's new eating style. As self-monitoring can enhance self-control and facilitate problem solving, patients will self-monitor their intake with a food diary, or by using an electronic tracking system. This will facilitate the counting of fiber grams in the high fiber condition, or other food components in the AHA condition. The dietitian will review and return food diaries to assist patients with meeting dietary goals. Dietitian providers are trained in a patient-centered counseling model and strategies from social-cognitive theory to activate patients to take action and responsibility for changing their dietary lifestyle to meet the goal prescribed [50]. All but the first group session will last 1 hour, and will include weighing the patient, a light snack of study appropriate foods, and other tools and methods (The first session lasts 1.5 hours). The first and last individual visits will be 1 hour, and 30 minutes respectively.

Group sessions will target individual food choices, environmental and social influences to dietary intake, lifestyle challenges, and problem-solving techniques in a supportive group format targeting dietary changes over the longer term. Significant others are invited to attend each session and to sign consent to have their weight tracked. Participants unable to attend a session will be offered brief make-up sessions, either by telephone, or in person and are mailed the materials from the missed session.

### High Fiber Diet Condition

Participants randomized to the high fiber condition receive instruction on how to gradually increase their dietary fiber intake to ≥ 30 g fiber per day, with a corresponding increase in non-caloric fluids as necessary to alleviate any gastrointestinal discomfort that may occur because of the increase in dietary fiber [51,52]. The multiple benefits of increasing dietary fiber will be outlined in an engaging, experiential format, with tasting of high fiber foods provided. Participants are encouraged to obtain fiber from a variety of high fiber foods, so they are not relying upon one type of food or fiber (such as high fiber bars or supplements), with a variety of recipes and substitutions suggested allowing for individual tastes, tolerance, and preferences. Participants receive written materials on the fiber content of different foods so they can choose from a vast list of foods that include both soluble and insoluble fiber, such as legumes, barley and other whole grains, nuts, seeds, fruit and vegetables. Self-monitoring will increase awareness and knowledge of intake to attain fiber goals. Participants will be working closely with the dietitian to ameliorate any intestinal discomfort (bloating, gas) associated with increasing fiber intake.

### AHA Diet Condition

Participants randomized to the AHA condition receive step-by-step instruction on the multiple components of dietary change, which includes a diet rich in vegetables and fruits; whole-grains, high-fiber foods; fish, especially oily fish at least twice a week; lean animal and vegetable proteins; learn to distinguish types of fats and oils; minimize intake of beverages and foods with added sugars; choose and prepare foods with little or no salt; and consume moderate to no alcohol intake. A target of 50-55% of calories from carbohydrate, 15-20% from protein, and 30-35% of calories from fat (saturated fat limited to <7% of energy, *trans*-fat to <1% of energy, cholesterol <300 mg/day,) will be suggested. Calorie goals will be calculated and provided to patients by estimating the daily calories needed to maintain the participant's starting weight and subtracting 500-1,000 calories/day (depending on initial body weight and level of activity) to achieve a 1-2 pound per week weight loss (i.e., (starting weight × 12) - 500 kcal). Self-monitoring will increase awareness and knowledge of dietary intake and progress toward AHA goals.

### Physical Activity

We acknowledge the beneficial effect of physical activity on weight loss and most metabolic syndrome components (elevated blood pressure, insulin resistance, and central obesity). However, the present study was designed to assess the effect of a simple dietary recommendation against the AHA diet on weight loss, overall dietary quality and factors of the metabolic syndrome. Therefore, we focus exclusively on dietary modifications during the sessions.

### Safety Precautions

Participants in both arms of the study will be advised to increase fiber slowly, and told to increase fluids to minimize digestive discomfort. Most participants will tolerate an increase in fiber, but some participants may experience gas, bloating, and changes to bowel habits. Participants will be monitored and encouraged to speak about any physiological or psychological difficulties of dietary change to allow the dietitian to assist with transitions to a higher fiber diet or AHA diet, both individually and as part of the group sessions. Over a short period of time (about 1 month or less), most digestive systems will adjust to an increase in dietary fiber. Occasionally, participants are unaware of diverticulosis, and dietary changes may trigger active disease. Participants with active diverticulitis and other gastrointestinal disease are ineligible. Additionally, there may be financial costs associated with making dietary changes, as the participants will be purchasing different foods.

### Training of Intervention Team Providers

All providers will be trained in the intervention delivery models relevant to their randomly assigned condition and will not be trained or have access to training materials for the other condition. Ample opportunities will be provided for developing and practicing the counseling skills to ensure fidelity to the intervention manual.

Four separate 2-hour training sessions are held. The training team includes the PI, a senior dietitian, and a clinical health psychologist. Dietitians will be oriented to the intervention manual for their condition, discuss difficult patient situations, review quality control procedures, and practice counseling skills. The team will work with each dietitian in typical patient interaction simulations. Lastly, each dietitian will present a selected "mock" session as a seminar at UMMS campus for faculty and students.

In order to provide the dietitian with feedback regarding their knowledge of the different interventions, all sessions are digitally recorded, with the senior dietitian listening to each of the first 5 sessions in order to provide feedback, and then 10% of sessions thereafter. Dietitian's knowledge, counseling and teaching skills will meet standards outlined on a certification checklist. Dietitians will attend regular supervision meetings with the senior dietitian, the clinical health psychologist, and PI to discuss study topics, fidelity to protocol, and group concerns.

### Treatment Fidelity

Because both conditions involve dietary interventions that have some subtle but very important differences, contamination and treatment fidelity will be closely monitored. Different dietitian providers are employed for each condition to prevent drift and/or contamination between conditions. During training and supervision, dietitians will be trained to handle situations when patients want to discuss topics not on protocol, with particular emphasis in the increase fiber condition to simply focus on consuming a variety of high fiber foods, without attention to distinction of calories, fats, carbohydrates or protein.

The purpose of fidelity monitoring is two-fold. First, we will ensure that treatment objectives for each condition are met at each session. Second, we will ensure that treatment objectives specific to one condition are not being met in the other condition (i.e., contamination). Two sets of treatment fidelity checklists (one arm-specific provider checklist and one auditor checklist) have been developed. Dietitians will complete the provider checklist after each session. A 10% randomly selected sample of the audio-recorded sessions will be reviewed by the senior dietitian and clinical psychologist. Each will then complete the Auditor Checklist corresponding to that session. When a session is reviewed with less than 85% of treatment-specific objectives met and/or any evidence of contamination (>0% other condition objectives met), the auditor will deliver that information to the PI who will then review the treatment objectives for that session with the counselor and remediate training as needed. This process will be ongoing throughout all treatment waves so that counselor drift can be swiftly corrected.

### Sample Size Consideration

The required numbers of patients for each arm were based on the primary outcome measure: change in body weight. Sample size was calculated using the method developed by Frison and Pocock [53] and implemented in Stata SE 10 (College Station, Texas, USA). Parameters used in sample size calculations were estimated in our pilot trial and historical data from metabolic patients in our clinics, and we assumed complete randomization of the study subjects to each treatment arms. With 95 complete cases per arm, the hypothesized difference in change in body weight (3.5 lbs) between the arms can be detected with >80% power at 5% significance level.

Considering possible attrition rate of 20%, the number of subjects in each arm should be not less than 120 subjects. Thus, a total of 240 patients will be enrolled in the study.

### Statistical Analytic Approach for Primary Aim

The high fiber condition is hypothesized to induce greater weight loss than the AHA condition. We will evaluate the intervention effects on change in body weight using general and generalized linear latent and mixed models (GLLAMM) that have been implemented in Stata SE 10 [54,55]. The commonly used linear mixed models are a subgroup of GLLAMM. In the analysis, each patient is assumed to be independent. First, we will carefully evaluate the covariance structure of the outcome variables across time points, and test whether intervention may induce changes in variability in the outcomes. Proper covariance structure will be identified and prescribed in the final analysis. The participant identifier will be included as random effect, time and group as fixed effect. Treatment effect will be tested using time*group interactions in the models.

The analysis will adjust for potential confounders for weight change, including age, gender, baseline BMI, marital status, education attainment, physician advice regarding weight loss and other nutritional advice exposure prior to and during the trial. We also will evaluate the possible interactions between the intervention indicator and these patient's attributes to assess the presence of differential intervention effects among these subgroups. Session attendance will also be included in the models to determine whether this is a dose-response relationship between number of session attended and the extent of weight loss. Statistical approaches to secondary outcomes are very similar, and thus not discussed in detail.

### Project Management and Participant Tracking

Under the direct leadership of the project director, project staff will be responsible for: 1) tracking patients to ensure that all necessary data are collected in a timely fashion; 2) assist with developing monitoring reports; 3) providing timely and relevant feedback to the leadership regarding the accuracy of data; and, 4) the day-to-day functioning of the study across all 10 waves of recruitment, intervention and assessment. The tracking system used for monitoring patient activities and providing necessary prompts based on a communication system using Lotus Notes from IBM (Lotus Notes R5.0.11^®^). Multiple levels of password protection are utilized to ensure data security. The tracking system will facilitate timely scheduling of assessments and identification of completed assessments.

### Data Entry and Management of Data Files

All data are entered into computerized data files (Epi Info for double-entry and Lotus Notes for patient tracking). All data entry systems employ automatic checks for values that are out of range or represent errors of faulty logic. Each patient will be assigned a study ID number to ensure confidentiality.

The data manager/programmer, under the supervision of the PI and project biostatistician will: 1) train project staff; 2) monitor all data collection protocols to assure compliance; 3) generate monitoring reports; 4) provide feedback regarding data accuracy and precision; and 5) implement variable edit checking. The study biostatistician will have responsibility for structuring the primary datasets, data linking procedures, variable naming conventions, codebooks, and documentation. Frequent exploratory analyses and range/value checking protocols will detect erroneous values. Data from each source will be merged using study-specific patient identification numbers and will be transferred to Stata SE 10 data files for analysis. All patient identifiers will be removed from analytic datasets. All database files will be stored on a password protected network drive with firewall protection that is managed by UMMS Information Service. All project-related data files will be automatically backed up daily per UMMS data safety protocol. A study directory will be established as the central repository for all final Stata datasets. Only the authorized project staff will have access to the databases. Project staff are prohibited to download data files with patient identifies to local drives unless authorized by the PI and Project Director.

## Discussion

Identifying a simpler dietary recommendation for weight loss and improving both dietary quality and metabolic health may demonstrate potential for a simple public health message to impact the metabolic syndrome and its sequelae of chronic disease. In a randomized clinical trial design, the present study will compare the efficacy of two intervention approaches to dietary change for CVD and diabetes prevention among persons with metabolic syndrome.

The prevalence of the metabolic syndrome is 26.7% according to the NHANES 1999-2000 survey of U.S. adults. Using 2000 census data, about 47 million US residents have the metabolic syndrome [4]. The metabolic syndrome is a harbinger of type 2 diabetes and CVD, both leading causes of mortality in the US. Lifestyle change is the cornerstone of recommended care for patients with metabolic syndrome [40,43].

Zivkovic and colleagues, in a comparative review of current dietary guidelines, diets, and dietary components, suggested that the AHA dietary recommendations are suitable for patients with the metabolic syndrome [41], with a similar suggestion made in a literature review, by Feldeisen and Tucker [42]. Theoretically, perfect adherence to the AHA diet would result in very high dietary quality and would impact components of the metabolic syndrome, however, progress in following this diet has been less than stellar. The composite of 13 different recommendations may be too complex for patients to understand and follow [6,9], limiting the benefits. The current study hypothesizes that it may not be necessary to give people guidance on every area of diet for diet quality to improve, because change in one area often result in changes in other areas of diet, both intentionally and unintentionally.

In a editorial on role of diet on insulin sensitivity and diabetes prevention, Xavier Pi-Sunyer recommended concentrating on educating the public to increase dietary fiber intake because "there is excellent evidence that the higher-fiber foods, made up of whole grains, fruits, and vegetables, will do people good"[20]. Although evidence demonstrates a link between dietary fiber, body weight and metabolic syndrome, more research is necessary to translate epidemiologic evidence and recommendations into effective clinical practice. To further this goal, the proposed study will measure inflammatory markers to elucidate possible mechanisms by which dietary fiber alone can impact metabolic syndrome, as compared to the more complex approach currently recommended by the AHA.

In addition to improving markers of metabolic syndrome, high intake of dietary fiber is associated with improved diet quality. In an observational study, Kranz and colleagues found that children in the high-fiber quartile consumed diets with higher nutrient density and increased number of servings from Food Guide Pyramid food groups (i.e.; fruit and vegetables, whole grains) [56]. Consumption of whole grain carbohydrates increased and the percentage of calories from fat significantly decreased; intake of micronutrients including iron, folate, vitamin A, and vitamin C increased significantly across quartiles of fiber consumption; and mean calcium intake also increased. In a review article, Lairon concluded that fiber-rich foods also have a number of bioactive phyto-chemicals that may have an additional beneficial role [12]. We analyzed the dietary quality of popular dietary plans and concluded that patients can lose weight with most dietary plans in the short term, but when the goal is to change the lifestyle and maintain changes, it is important to consider whether or not the diet is healthful for cardiovascular concerns over the long-term [57]. This is especially true for patients with metabolic syndrome. Our findings suggest that the highest fiber diets were the best in terms of diet quality.

By focusing on a single aspect of diet - increasing dietary fiber - patients may choose foods of higher dietary quality (whole grains, fruit, vegetables, legumes) without feeling overwhelmed by the complexity of multiple dietary changes. Increased dietary quality and fiber intake were inversely association with body weight in previous studies ([10-12,58]. Our preliminary work reveals that increases in fiber may be associated with changes in other aspects of diet (e.g., reducing saturated fat intake), thereby affecting dietary quality even beyond the simple increase in fiber intake. We concluded from our pilot study that it may not be necessary to give instruction on several areas of the diet because correct simple changes might beneficially influence other areas of diet [39]. Simplifying diet changes, increasing fiber intake appears to have excellent effects on weight and factors of the metabolic syndrome.

## Conclusion

Data from the present study will enhance our understanding of the overall impact of a simple dietary change on metabolic health and diet. If the simple fiber approach is successful, it may then be used to develop a simple public health message.

## Competing interests

The authors declare that they have no competing interests.

## Authors' contributions

YM, SP, WL, BO, IS, KS and PM participated in conception, and design of the study. YM and PM drafted the manuscript, PM, SP, WL, BO, IS, and KS critically revised the manuscript and all authors read and approved the final manuscript.

## Pre-publication history

The pre-publication history for this paper can be accessed here:

http://www.biomedcentral.com/1471-2288/9/87/prepub
